# A Luciferase Gene Driven by an Alphaherpesviral Promoter Also Responds to Immediate Early Antigens of the Betaherpesvirus HCMV, Allowing Comparative Analyses of Different Human Herpesviruses in One Reporter Cell Line

**DOI:** 10.1371/journal.pone.0169580

**Published:** 2017-01-06

**Authors:** Anna Katharina Maier, Raimund Jung, Clarissa Villinger, Axel Schubert, Paul Walther, Christian Sinzger, Diana Lieber

**Affiliations:** 1 Institute of Virology, Ulm University Medical Center, Ulm, Germany; 2 Central Facility for Electron Microscopy, Ulm University, Ulm, Germany; University of St Andrews, UNITED KINGDOM

## Abstract

Widely used methods for quantification of human cytomegalovirus (HCMV) infection in cell culture such as immunoblotting or plaque reduction assays are generally restricted to low throughput and require time-consuming evaluation. Up to now, only few HCMV reporter cell lines have been generated to overcome these restrictions and they are afflicted with other limitations because permanently expandable cell lines are normally not fully permissive to HCMV. In this work, a previously existing epithelial cell line hosting a luciferase gene under control of a Varicella-zoster virus promoter was adopted to investigate HCMV infection. The cells were susceptible to different HCMV strains at infection efficiencies that corresponded to their respective degree of epithelial cell tropism. Expression of early and late viral antigens, formation of nuclear inclusions, release of infectious virus progeny, and focal growth indicated productive viral replication. However, viral release and spread occurred at lower levels than in primary cell lines which appears to be due to a malfunction of virion morphogenesis during the nuclear stage. Expression of the luciferase reporter gene was specifically induced in HCMV infected cultures as a function of the virus dose and dependent on viral immediate early gene expression. The level of reporter activity accurately reflected infection efficiencies as determined by viral antigen immunostaining, and hence could discriminate the cell tropism of the tested virus strains. As proof-of-principle, we demonstrate that this cell line is applicable to evaluate drug resistance of clinical HCMV isolates and the neutralization capacity of human sera, and that it allows comparative and simultaneous analysis of HCMV and human herpes simplex virus type 1. In summary, the permanent epithelial reporter cell line allows robust, rapid and objective quantitation of HCMV infection and it will be particularly useful in higher throughput analyses as well as in comparative analyses of different human herpesviruses.

## Introduction

Human cytomegalovirus (HCMV) is a betaherpesvirus that persists lifelong in the host after primary infection. The pathogenic potential of HCMV becomes apparent in immunocompromised individuals such as transplant recipients or AIDS patients, where an overwhelming reactivation of the virus can cause life-threatening conditions. Effective antiviral drugs such as ganciclovir (GCV) or foscarnet (FOS) are available, however, they target mostly the same step in the viral replication cycle, which is DNA amplification by the viral DNA polymerase, and they are frequently counteracted by resistance-inducing mutations [1–4]. Therefore, continued research is required to better understand the molecular mechanisms of infection and to identify potential new drug targets and antiviral agents.

For these purposes, recombinant viruses have been generated that carry reporter genes encoding fluorescent proteins or proteins with enzymatic functions in order to allow straightforward and quantitative monitoring of viral infection [5–13]. Reporter viruses have for example been used (i) to study genotypic variants conferring drug resistance in a standardized genetic background [5,7], (ii) to identify or investigate antiviral substances [6,11,13,14] or (iii) to analyze the neutralization capacity of antibodies [8,10,15]. These approaches show the usefulness of reporter genes to study a wide range of different aspects but obviously, one-by-one modification of viral genomes is required and the examination of recent clinical isolates is excluded.

Until now, few HCMV reporter cell lines have been established as cell-based assay systems to overcome these limitations. In most cases, reporter genes controlled by HCMV promoters were inserted into the HCMV-susceptible human glioma cell line U373-MG [16–18] or in mink lung cells [19]. Either firefly luciferase [16,17] or green fluorescent protein (GFP) [18,19] have been chosen as reporters in these studies. Different HCMV early promoters were used to control reporter gene expression: pUL54 [17–19], pUL112/113 [18] or pTRL4 [16]. The promoters have in common that they are activated only by HCMV infection and not by infection with human alpha- or other betaherpesviruses (herpes simplex virus type 1 and 2 [17–19]; Varicella-zoster virus [16,19]; human herpesvirus type 6 [16]). This high level of specificity is useful in diagnostic applications where multiple herpesviruses in the same patient sample need to be distinguished. However, a reporter cell line that is susceptible and responsive to different closely related virus species would be advantageous in fundamental research as it allows comparative studies in the same assay system.

Another reporter cell line established by Ueno and colleagues in the background of Chinese hamster ovary (CHO) cells reports HCMV infection by the re-localization of a cellular GFP-fusion protein from the PML-bodies towards a pan-nuclear localization pattern [20]. The common principle of this and the above mentioned reporter cell lines is the sensing of viral immediate early functions. The need for this arises from the fact that HCMV infection does not proceed beyond the immediate early phase in CHO cells [20,21] similar to most other permanent cell lines. This restriction limits the use of existing HCMV reporter cell lines to the analysis of initial infection events and emphasizes the need for a reporter cell line that allows HCMV to complete its replicative cycle.

In this work, a pre-existing heterologous reporter cell line of human epithelial origin [22] is characterized as a cell-based assay system for quantitative analysis of HCMV infection. The cells are susceptible to HCMV and interestingly allow productive infection and viral spread. HCMV infection induces reporter gene expression which is in contrast to a remark in a previous study and therefore somewhat surprising. Moreover, the reporter gene responds to infection by different HCMV strains, including clinical isolates and to human herpes simplex virus type 1 (HSV-1) in a dose-dependent manner. The cell line can be applied to evaluate inhibition of different steps of the replicative cycle as is shown exemplarily for viral entry and DNA replication and to comparative analysis of herpesviruses from different subfamilies. Together, the study adds a cell-based reporter system for HCMV that has unique advantages over existing reporter systems and that can be useful in fundamental research as well as in diagnostic applications.

## Materials and Methods

### Cell culture

Human foreskin fibroblasts (HFF) were cultured in MEM supplemented with GlutaMAX (Life Technologies), 5% fetal bovine serum, 0.5 ng/ml basic fibroblast growth factor (bFGF) and 100 μg/ml gentamicin (Life Technologies). HEC-LTT cells originate from human umbilical vein endothelial cells that contain doxycycline-inducible expression cassettes for SV40 large T antigen and the human telomerase catalytic subunit [23], and were maintained in endothelial growth medium (EGM BulletKit, Lonza) with 2 μg/ml doxycycline (Sigma-Aldrich) culture vessels coated with 0.1% gelatin for 30 min (Sigma-Aldrich). Infection was performed 1 d after doxycycline withdrawal [24]. MV9G cells [22] were cultured in minimum essential medium (MEM) supplemented with GlutaMAX (Life Technologies), 10% fetal bovine serum (PAA), 50 μg/ml G-418 (GE Healthcare) and 100 μg/ml gentamicin (Life Technologies) as previously described [25]. The human melanoma cell line MeWo [26–28] (ATCC-HTB-65) was purchased from LGC Standards. MeWo and Vero cells [29] (obtained from ATCC) were cultured in Dulbecco's Modified Eagle's medium supplemented with GlutaMAX (Life Technologies), 10% fetal bovine serum (PAA) and 100 μg/ml gentamicin (Life Technologies).

### Virus strains and stocks

HCMV strains TB40/F and TB40/E originate from a single isolate from a bone marrow transplant patient and were passaged in HFF or in endothelial cells, respectively [30]. Similarly, VHL/E and VHL/F have been generated from a common parental isolate by propagation in endothelial cells or fibroblasts, respectively [31]. AD169 [32] and Towne [33] are well-characterized laboratory-adapted strains with restricted cell tropism. Cellular debris was removed to generate virus stocks by centrifugation of supernatant from infected HFF cultures at 5 to 7 d p.i. (days post infection) at 3,345 X *g* for 10 min. Aliquots were stored at -80°C until use. Titers of infectivity were determined in HFF by serial dilution assays.

HSV-1 strain F was propagated in Vero cells. To generate stocks, supernatants of HSV-1 (F) infected cells were harvested at 3 d p.i. and cellular debris was removed by centrifugation at 3,345 X *g* for 10 min. Stocks were stored at -80°C until use.

### Detection of viral antigens

Indirect immunofluorescence analysis was performed to detect immediate early (IE) antigens 1 and 2 (pUL122/123). Cells were fixed at 24 h p.i. with 80% acetone for 5 minutes at ambient temperature (Sigma-Aldrich). The primary mouse antibody E13 (Argene) against viral IE antigens 1 and 2 was used and visualized by a Cy3-conjugated goat polyclonal anti-mouse-Ig F(ab´)_2_ antibody (Jackson Immunoresearch) or an Alexa Fluor 488-conjugated goat anti—mouse-Ig F(ab’)_2_ antibody (Life Technologies) as secondary antibodies. Nuclei were counterstained with DAPI (Sigma Aldrich). Fluorescence images were taken with an Axio Observer D1 microscope (Zeiss) and Cy3-positive nuclei as well as DAPI-stained nuclei were counted. The rates of IE antigen-expressing cells representing infection efficiencies were calculated for each image and normalized to the respective control. For assays performed in 96-well plates, two replica wells per condition were prepared, three images were taken per well and mean values were calculated.

For indirect immunostaining of early (pUL44) and late (major capsid protein, MCP) viral antigens, cells were fixed with 80% acetone at 1 or 5 d p.i. and incubated with mouse hybridoma supernatant clone 5.4 for pUL44 (Biotest AG) and clone 28–4 for MCP (kindly provided by W. Britt, University of Alabama at Birmingham). A Cy3-conjugated goat polyclonal anti-mouse-Ig F(ab')_2_ antibody (Jackson ImmunoResearch) was used as the secondary antibody and nuclei were counterstained with DAPI.

### Plasmids, siRNA and transfection

Plasmid pRR47 [34] was a kind gift from Bodo Plachter, Mainz. The control plasmid pmCherry-C1 encodes mCherry under control of a major immediate early promoter of HCMV and was kindly provided by Kerstin Laib Sampaio, Ulm. Plasmid transfection was performed with TurboFECT reagent (Thermo Fisher Scientific) according to the manufacturer’s instructions. Transfection was normalized by using the same total amount of plasmid and transfection reagent for each condition. Transfection efficiency was between 30% and 50% as estimated by fluorescence microscopy (mCherry) or immunofluorescence analysis (IE expression) of replica cultures.

siRNA transfections were performed as previously described [35]. A non-targeting siRNA pool (siGENOME Non-Targeting Pool #2, Thermo Fisher Scientific) and an siRNA effectively targeting the HCMV immediate early antigens 1 and 2 (UL122/123) (5’-CCGAGGAUUGCAACGAGAA[U][U]-3’, Sigma-Aldrich) [35] were reversely transfected in MV9G cells in 96-well plates with Lipofectamine RNAiMAX (Life Technologies) according to the manufacturer’s instructions. Transfections were performed in five replicates. Cells were seeded at a density of 1x10^4^ cells per well, and the final siRNA concentration was 50 nM. Infection with HCMV TB40/E or HSV-1 (F) was performed 2 d post transfection. Infection efficiency of HCMV was analyzed at 1 d p.i. by immunodetection of viral immediate early antigens in two replicates and luciferase activity was measured in the remaining triplicates after cell lysis at 2 d p.i. as described below. When comparing HCMV- and HSV-1-dependent luciferase activity in the same experiment, the luciferase assay was performed at 1 d p.i. because of the short replication cycle of HSV-1.

### HCMV cell tropism analysis

Tropism assays were performed as previously described [24]. Briefly, cells were seeded 1 day prior to infection at a density of 1x10^4^ cells/well in duplicates in 96-well plates. For HEC-LTTs, wells were coated with 0.1% gelatin before adding cells. All cells were incubated in MEM supplemented with GlutaMAX (Life Technologies) and 5% fetal bovine serum (termed MEM5) for 30 min prior to infection with serial dilutions of the respective HCMV strains in MEM5. At 2 h p.i., the medium was replaced by cell type-specific medium. At 1 d p.i., expression of immediate early antigens 1 and 2 (pUL122/123) was detected by indirect immunofluorescence as described above to yield infection efficiencies. In order to determine the relative tropism, the infection efficiency of a virus strain in each cell type was related to the infection efficiency in HFF.

### Documentation of cytopathic effects

The different cell types were seeded at a density of 1x10^5^ cells/well in 6-well plates 1 day prior to infection. Infection was performed with fresh infectious supernatant of cultures infected with HCMV strain TB40/E for 5 days. Prior to infection, virus-containing supernatant was cleared of cellular debris by centrifugation at 3,345 X *g* for 10 min. The medium was renewed daily and the cell morphology documented by phase contrast microscopy using a Zeiss Axio Observer D1 microscope.

### Quantification of infectious virus release

The experiments were essentially performed as described previously [24]. Briefly, the different cells were seeded in 6-well plates at 1x10^5^ cells/well. One day after seeding, the cells were incubated in MEM5 for 30 min prior to infection and infected overnight with serial dilutions of HCMV TB40/E in MEM5. Replicate wells were prepared and analyzed at 1 d p.i. by immunostaining of viral immediate early antigens. Those wells were identified that yielded comparable infection efficiencies for all cell types (>63% infected cells) and their respective replicate cultures were selected for further analysis. The cultures were washed four times with fresh medium and the last aliquot was saved for determination of background infectivity. All samples were cleared of cellular debris by centrifugation at 3,345 X *g* for 10 min before storage at -80°C. Culture supernatants were harvested at 5 and 7 d p.i. All samples were analyzed in parallel for infectious titers by serial dilution assays on HFF.

### Quantification of viral genome replication

Quantification of viral DNA in infected cells and in supernatants of infected cultures was performed as previously described [24]. Briefly, cells were mock infected or infected for 2 h with strain TB40/E. The virus dose was adjusted to achieve comparable infection of all cell types. Supernatants and cells were harvested at 0 (mock), 8 and 16 h p.i. and at 1, 2, 3, 5 and 7 d p.i. and stored at -80°C until processing. DNA was extracted with the QIAamp Blood Mini Kit (Qiagen, Hilden, Germany) according to the manufacturer’s instructions. Quantification of viral DNA was performed by real-time PCR assays as described in [36]. To determine the ratio of viral genomes per infectious unit in the supernatants at 5 and 7 d p.i., aliquots of the same culture supernatants used for DNA quantification were titrated on HFF by serial dilution assay and the titers of infectivity were calculated.

### Transmission electron microscopy (TEM)

For comparative analysis of the late stage of HCMV infection in HFF and MV9G cells we conducted TEM. To provide optimal visibility of the viral and cellular ultrastructure, the methods of high-pressure freezing, freeze substitution, and Epon embedding were performed as previously described [37]. For that, HFF and MV9G cells were seeded in their respective medium on carbon-coated sapphire discs (3 mm in diameter, 50-μm thickness; Engineering Office, M. Wohlwend GmbH), which were placed into μ-Slide eight-well plates (Ibidi GmbH). After adhesion of cells on sapphire discs for 24 hours, cells were infected with TB40/E with an MOI around 2. At 5 d p.i., cells on sapphire discs were fixed by high-pressure freezing (Compact 01 high-pressure freezer, Engineering Office, Wohlwend GmbH), subjected to freeze substitution [38,39] and subsequently embedded in Epon (Fluka). For TEM, ultrathin sections were cut with an ultramicrotome (Ultracut UCT; Leica), mounted onto Formvar-coated single-slot grids (Plano GmbH), and examined in a JEM-1400 transmission electron microscope (Jeol) equipped with a CCD camera at an acceleration voltage of 120 kV. Only cells that showed the highest densities of capsids inside the nucleus were analyzed on each ultrathin section to ensure that the analyzed cells were in similar stages of virion morphogenesis.

### Analysis of luciferase activity upon HCMV infection

MV9G cells were seeded in 96-well-plates at 5x10^4^ cells per well and infected after 1 day with serial dilutions of HCMV strains TB40/E, TB40/F or Towne. Five replicates were prepared per condition. At 1 d p.i., duplicates were fixed with 80% acetone and immunostained to detect viral immediate early antigens 1 and 2 as described above. The fraction of infected cells was calculated. The remaining triplicates were subjected to cell lysis at 2 d p.i. with Passive Lysis Buffer (Promega). The lysates were diluted 1:5 with phosphate buffered saline and one tenth of each lysate was analyzed for luciferase activity using the Luciferase Assay System (Promega) as previously described [25]. The measurement was performed in a Chameleon plate reader with automatic injection system (Hidex).

### Neutralization assay

MV9G cells were seeded at a density of 5x10^4^ cells/well in 96-well plates. For evaluation of the neutralization capacity of the glycoprotein B (gB) antibody C23 (kindly provided by Michael Mach; [40,41]), serial dilutions of the antibody were prepared in MEM5 and mixed with HCMV TB40/E in MEM5. The virus dose was adjusted to achieve 50–70% infection. The mixture was incubated for 1 h at ambient temperature before addition to the cells. The inoculum was removed after 2 h and replaced with fresh medium. In order to control the infection dose, cells were infected in parallel in absence of gB antibody. Five replicates were prepared and two of them were subjected to immunostaining of immediate early antigens at 1 d p.i. The remaining three replicates were subjected to cell lysis and luciferase assay at 2 d p.i. as described above.

Similarly, the HCMV neutralization capacity of human sera was determined by incubating virus with serial dilutions of serum prior to infection in replicate cultures followed by immunostaining of viral immediate early antigens (one replicate well, three images per well) and luciferase assay (two replicate wells).

The concentration of antibody or serum causing a 50% reduction of either luciferase activity or the fraction of infected cells (50%-neutralization dose, ND50) was determined by fitting of a sigmoidal dose response curve to the measured values plotted against the logarithmic dilution (SigmaPlot, SystatSoftware Inc.).

### Comparative analysis of HCMV and HSV-1

Human sera were used that were seropositive for either HCMV or HSV or both. HCMV-IgG and HSV-IgG concentration was determined by CMV-IgG-ELISA PKS (Medac, Germany) or Enzygnost Anti-HSV/IgG (Siemens, Germany), respectively. The IgG indices (HSV-IgG / HCMV-IgG index) of the selected sera were as follows: 0.22 / 26.7; 20.04 / 0; 17.85 / 24. 2x10^4^ MV9G cells per well were seeded on 96-well microtiter plates and incubated for 2 days at 37°C. Dilutions of the human sera in MEM5 supplemented with 100 μg/ml gentamicin were mixed with either HSV-1 or HCMV or both. The human sera were used at a 1:30 dilution in order to ensure efficient virus neutralization. The virus doses were adjusted to yield similar levels of luciferase activity within the respective linear range of the virus. After 1 h incubation at 37°C, the serum/virus mixtures were added to the cells in triplicates. To control for the background luciferase, triplicate wells were incubated with medium only. In parallel, cells were infected with virus that was preincubated in medium without human serum in order to determine the maximum luciferase activity induced by each virus or the virus combination. After 1 h infection at 37°C, the serum/virus mixtures were removed from the cells and replaced by medium. Cells were lysed 1 d p.i. and subjected to luciferase assays as described above.

### Focus expansion assay

Focus formation assays were in principle performed as previously described [42]. Briefly, MV9G or MeWo cells were co-cultured with declining numbers of TB40/E-infected cells in quadruplicates and incubated for 7 days to allow for focus formation. Cultures were fixed with 80% acetone and viral immediate early antigens were detected by indirect immunofluorescence. The number of infected cells per focus was counted in co-cultures where about 10 foci defined as at least three infected adjacent cells had formed. The focus expansion (FE) value was determined as described [42].

### Drug resistance assay

HFF uninfected or infected with recent clinical isolates were co-cultured in 96-well plates to yield 30 foci per well on average. The infection was allowed to progress in presence of increasing concentrations of either ganciclovir (0.4–400 μM) or foscarnet (12.5–1600 μM) for 5 days. 3x10^4^ MV9G cells per well were added and allowed to settle and receive virus for two days. Then, the cells were lysed and subjected to one freeze-thaw cycle at -20°C before measuring the luciferase activity. In order to determine the inhibition, the mean luciferase signals from four replicate wells were calculated, normalized to the highest mean obtained with the respective isolate and subtracted from 1. The effective drug concentration leading to 50% inhibition (EC50) was determined by fitting of a sigmoidal dose response curve to the inhibition values plotted against the drug concentration (SigmaPlot, SystatSoftware Inc.).

## Results

The epithelial reporter cell line MV9G is derived from the human melanoma cell line MeWo and carries a firefly luciferase gene under control of a Varicella-zoster virus (VZV) ORF9 promoter fragment [22]. First, we tested whether MV9G cells are in principle susceptible to HCMV infection with a virus strain known to be highly epitheliotropic (TB40/E) and several poorly epitheliotropic (TB40/F, AD169 and Towne) virus strains that are frequently employed as laboratory strains. The restricted cell tropism of the three poorly epitheliotropic strains is due to different mutations affecting components of the pentameric glycoprotein H/L complex (gH/gL/pUL128/pUL130/pUL131A): UL128 in TB40/F, UL131A in AD169 and UL130 in Towne [43–47].

**Fig 1:**
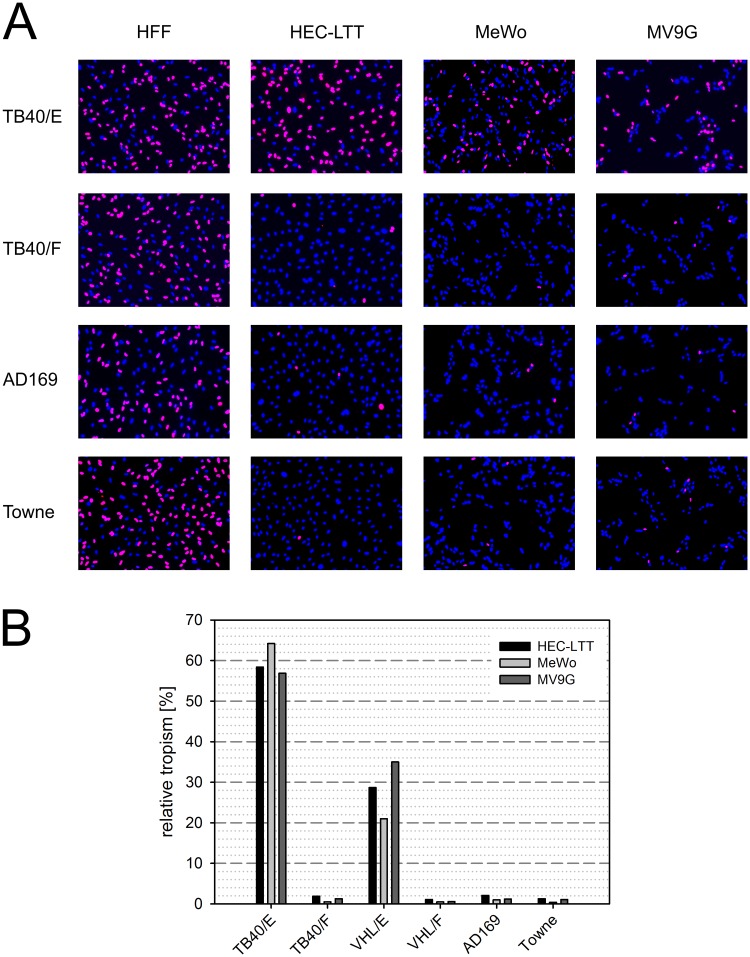
MV9G cells are in principle susceptible to epitheliotropic virus strains. (A) Indicated cells were infected with different laboratory strains of HCMV at a virus dose yielding approximately 60–70% infected cells in HFF. The infection efficiency was visualized by immunostaining of viral immediately early antigens (red) and the nuclei were counterstained with DAPI (blue). (B) The cells were infected with serial virus dilutions and the fractions of infected cells were determined by immunostaining. The relative tropism of each virus strain compared to the reference cell type HFF is given in percent. To calculate the relative tropism, data from three independent experiments was combined.

To test susceptibility to these HCMV strains, MV9G cells were seeded in 96-well plates and infected with a virus dose that yields 60–70% infection in HFF. As a control for the virus dose, human foreskin fibroblasts (HFF) were included in each experiment. Conditionally immortalized human umbilical vein endothelial cells (HEC-LTT) [23,24] and the parental epithelial cell line of the MV9G cells, MeWo, were used to control for the broad or restricted cell tropism of the virus strains. Infected cells were visualized by immunofluorescence detection of viral immediate early (IE) antigens (pUL122/123). As expected, HFF were readily infected by all strains, while the strains with restricted cell tropism (TB40/F, AD169 and Towne) failed to efficiently express IE antigens in the reference endothelial and epithelial cells (Fig 1A). Accordingly, TB40/E infected MV9G cells at a higher efficiency compared to the other virus strains.

In order to analyze the susceptibility of MV9G cells in a quantitative manner, the cells were infected with serial dilutions of all four viruses and the fractions of infected cells were determined by immunofluorescence staining. Two more virus strains with known broad (VHL/E) or restricted cell tropism (VHL/F) were included [31]. The relative cell tropism was calculated by relating the infection efficiencies of the viruses in each cell type to the efficiency in HFF. Each virus yielded a similar relative tropism in MV9G cells as in MeWo and HEC-LTT cells (TB40/E 57–64%, VHL/E 21–35%, poorly epitheliotropic strains ≤ 2%) (Fig 1B).

Taken together, MV9G cells were in principle susceptible to HCMV and accurately reflected the expected broad or restricted cell tropism of several virus strains.

**Fig 2:**
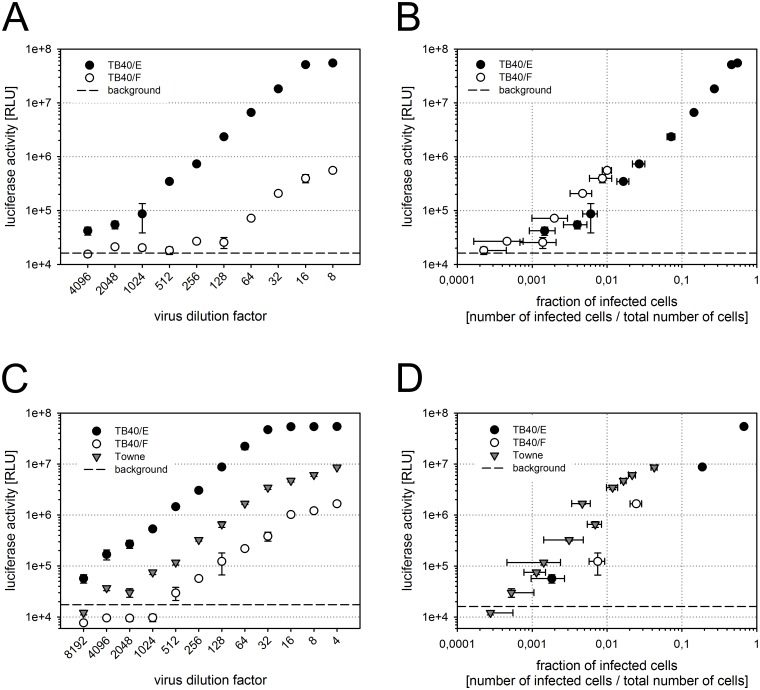
The luciferase gene accurately reports the HCMV infection efficiency. MV9G cells were infected with serial dilutions of virus strains TB40/E and TB40/F (A-D) or Towne (C-D). The luciferase activity (RLU, relative light units) was measured and plotted against the dilution of the virus preparation (A, C) or against the fraction of infected cells which was determined by immunodetection of viral immediate early antigens in parallel cultures (B, D). The background signal was determined in uninfected MV9G cells. Each graph shows data from one representative experiment out of four (A-B) or two (C-D). Error bars: SEM.

Next, we investigated whether the heterologous luciferase reporter gene of the MV9G cells responds to HCMV infection. The luciferase activity was measured after infection of MV9G cells with serial dilutions of the virus strains TB40/E and TB40/F. In parallel cultures, infected cells were detected by immunofluorescence staining of viral immediate early antigens in order to compare the reporter activation capacity of the highly and the poorly epitheliotropic strain. In principle, both virus strains activated the reporter gene at detectable levels (Fig 2A). The luciferase activity could be discriminated from the background even at less than 1% infected cells in the culture (Fig 2B). The level of the luciferase activity increased with the virus dose and this was observed with both virus strains, TB40/E and TB40/F (Fig 2B). To confirm this, we tested another poorly epitheliotropic strain, Towne, and observed a similar capacity to induce reporter gene activity as with TB40/E and TB40/F (Fig 2C and 2D). Thus, the luciferase activity reported the infection efficiency in a quantitative manner, accurately and as a function of the viral cell tropism.

**Fig 3:**
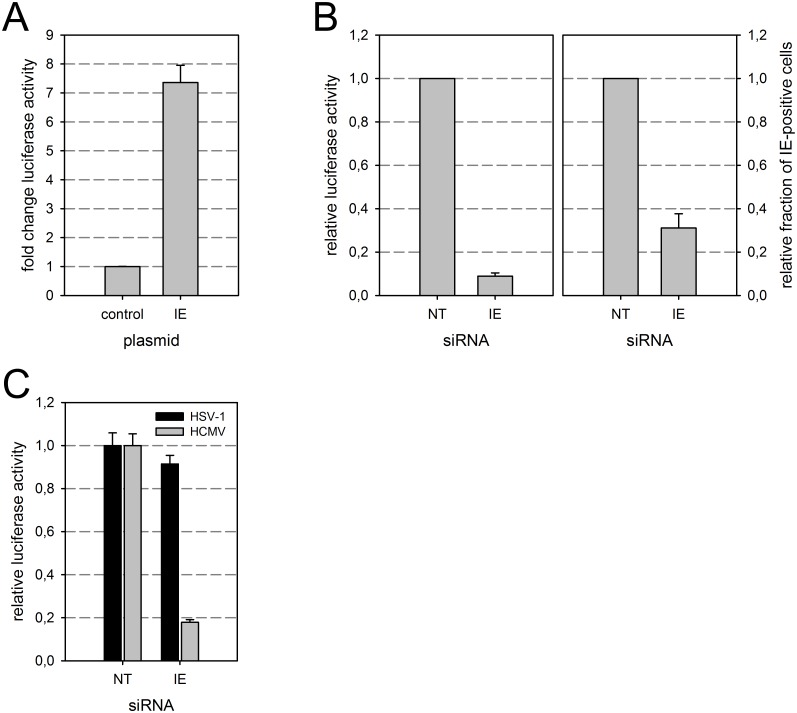
HCMV IE antigen expression is required and sufficient to activate the heterologous reporter gene. (A) MV9G cells were transiently transfected with a control plasmid (pmCherry-C1) or an immediate early antigen expression plasmid. The luciferase activity was measured. (B-C) MV9G cells were transfected with non-targeting (NT) siRNA or siRNA targeting the viral immediate early antigens UL122/123 (IE). After 2 days, cells were infected with HCMV TB40/E (B-C) or HSV-1 (C) and luciferase activity was measured after another 2 days (B, left) or at 1 d p.i. (C). In replicate cultures, the efficiency of the IE siRNA was controlled by immunodetection of immediate early antigens at 1 d p.i. (B, right). The data is normalized to the respective control. (B) The graph summarizes data from three independent experiments. (C) One representative experiment is shown (n = 3). Error bars: SEM.

It has been shown previously that VZV IE62 is sufficient to induce the reporter gene expression in co-transfection experiments [22]. To date, no orthologue of VZV IE62 is known in betaherpesviruses [48]. In order to investigate how the reporter gene is activated in HCMV infection, we transiently transfected a plasmid containing the genomic HCMV immediate early UL122/123 gene region encoding the major immediate early transactivators IE1 and IE2 into MV9G cells and measured luciferase activity. In the UL122/123-transfected cultures, luciferase activity was increased by more than 7-fold compared to cultures transfected with a control plasmid (Fig 3A). This suggests that expression of viral immediate early genes was sufficient for reporter gene activation in absence of infection. To exclude that components of the incoming virion or a cellular process induced by HCMV entry contribute to reporter gene activation, MV9Gs were transfected with a short interfering RNA (siRNA) directed against both viral immediate early antigens UL122 and UL123 [35,49] or a non-targeting control siRNA prior to infection with the virus strain TB40/E. The luciferase activity was reduced in UL122/123 siRNA-treated cells by 91% ± 2 SEM (Fig 3B, left). The function of the siRNA was controlled by immunodetection of UL122/123 in parallel cultures which showed a reduction of IE-expressing cells by 69% ± 7 SEM (Fig 3B, right). The UL122/123 siRNA acted stronger on the luciferase activity than on the UL122/123 antigen abundance. This fits with the observation that the UL122/123 immunofluorescence staining in the culture treated with UL122/123 siRNA was generally weaker than in the control culture (data not shown), probably because viral antigen expression is partially suppressed in cells with less efficient ratios of siRNA and virus. In consequence, cells expressing low amounts of UL122/123 contributed more to the antigen positivity than to the luciferase activity.

Reporter gene activation by human herpesvirus type 1 (HSV-1) was not significantly affected by HCMV UL122/123 siRNA which further argues against an unspecific effect of the siRNA on the reporter gene (Fig 3C). Together, the data indicated that HCMV immediate early gene expression was required in presence of HCMV infection and sufficient in absence of infection to activate the heterologous luciferase reporter gene.

**Fig 4:**
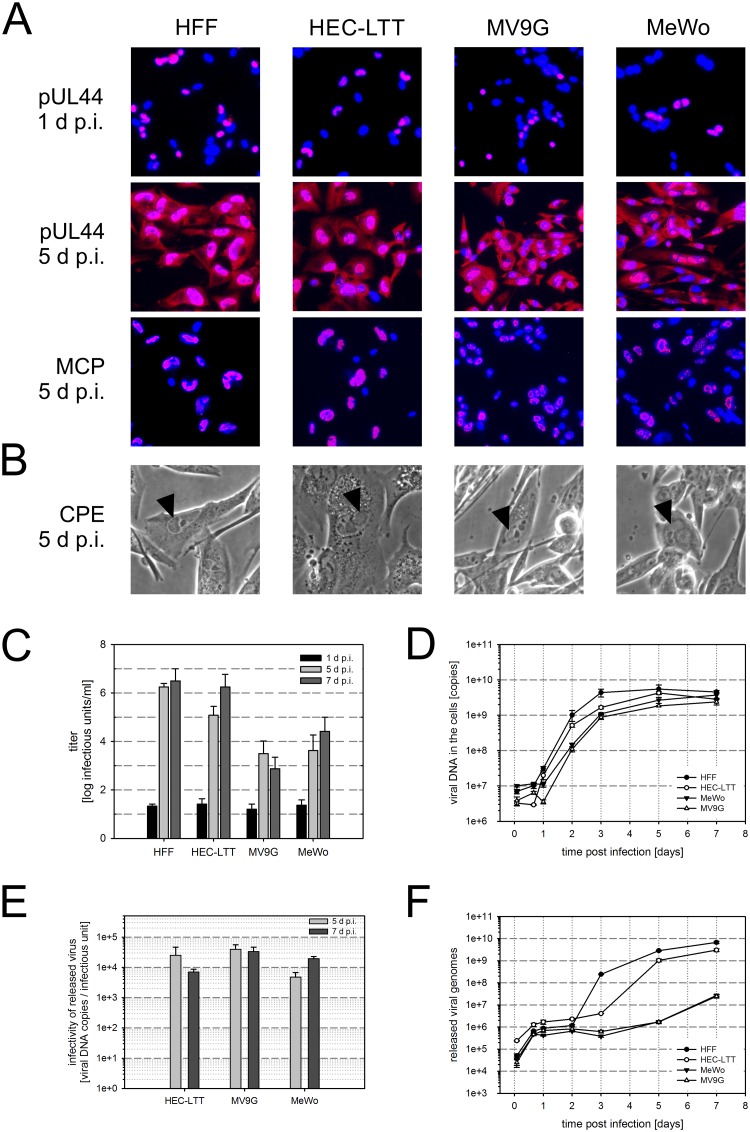
HCMV replicates efficiently in MV9G cells. (A) The indicated cells were infected with HCMV TB40/E, fixed after 1 or 5 d p.i. and immunostained for viral antigens pUL44 or MCP (red). Nuclei were counterstained with DAPI (blue). (B) Microscopic images of cells showing cytopathic effects (CPE) were taken at 5 d p.i. using phase contrast microscopy. The arrows indicate nuclear inclusions. (C) Supernatants of TB40/E-infected cell cultures were harvested at 1, 5 or 7 d p.i. and analyzed by serial dilution assay on HFF to determine the titers of infectivity. (D-F) Cells and supernatants of TB40/E-infected cell cultures were harvested at the indicated times. The DNA was extracted and the viral genomes were measured by quantitative RT-PCR. (D), copy number of viral DNA in the cell; (F), copy number of viral DNA released into the supernatant. (E) Supernatants of cultures infected for 5 and 7 days with TB40/E were harvested and titrated in HFF. The genome copy number per ml that was determined in the same supernatants by quantitative RT-PCR was divided by the titer (infectious units per ml) to determine the ratio of genome copies per infectious unit. Error bars: SEM (C, D, F); SD (E).

Quantification of viral growth in a cell culture over more than one replication cycle requires efficient viral replication and spread. Only few permanent cell lines are productively infected by HCMV, including HEC-LTTs [24,50–53]. In order to test whether HCMV infection proceeds in MV9Gs beyond the immediate early phase, expression of viral antigens representative of the early (pUL44) and late (major capsid protein, MCP) kinetic class of proteins was investigated. MV9G and MeWo cells as well as HFF and HEC-LTT cells were infected with HCMV strain TB40/E, fixed at 1 or 5 d p.i. and subjected to immunofluorescence staining. At 1 d p.i., pUL44 was similarly detectable in all four cell types and mostly localized to the nuclei (Fig 4A). The staining intensity was increased at 5 d p.i. and the localization of the signal was nuclear and cytoplasmic. MCP was readily detectable in MV9Gs, as in all other cell types, in confined regions within the nuclei that were indicative of sites of viral replication. In accordance with the MCP staining pattern, nuclear inclusions were observed by means of phase contrast microscopy at 5 d p.i. in MV9G and MeWo cells similar to HFF and HEC-LTT cells (Fig 4B). Together, these data suggest that HCMV infection reaches the late phase of the replication cycle in MV9G and MeWo cells and that viral infection progresses similarly to HFF and HEC-LTT cells.

To test whether infectious progeny virus is released from MV9G and MeWo cells in comparison to HFF and HEC-LTT cells, supernatants of cultures infected with HCMV TB40/E at comparable infection rates were collected at 1, 5 and 7 d p.i. Infectivity in the supernatants was quantified by serial dilution assay in HFF. At 5 and 7 d p.i., the titers of infectious virus were markedly increased in all supernatants compared to the background titers measured at 1 d p.i. (Fig 4C). Thus, all cell types had released infectious progeny virus, albeit the titers obtained from MV9G and MeWo cultures were 2–3 log steps lower than the titers in HFF and HEC-LTT supernatants.

This difference might be either due to a reduced virus replication, or to a less efficient virus release, or to diminished infectivity of the released progeny virus. In order to examine this, viral DNA in the cell and released viral genomes were monitored by quantitative RT-PCR in infected cell cultures over 7 days and the infectivity in the supernatants of the infected cultures was determined by titration on HFF (Fig 4D–4F). Overall, MV9G and MeWo cells contained comparable amounts of viral DNA in the cells as HFF and HEC-LTT cells at 5–7 d p.i. with a slight delay during the first 3 days of infection (Fig 4D–4F). In contrast, the amount of viral genomes in the supernatant of MV9G and MeWo cells was up to 3 log steps lower than in HFF or HEC-LTT cultures (Fig 4F), while the copy number per infectious unit was similar between MV9G, MeWo and HEC-LTT cells (Fig 4E). The difference in the amount of viral DNA in the supernatant correlates well with the degree of the observed difference in released infectivity. In conclusion, the morphological phenotype of infected cells, the expression of viral antigens of different kinetic classes and the viral DNA replication in the cell appear similar in MV9G and MeWo cells as in prototypic fibroblasts and endothelial cells, however, release of progeny virus from these cells seems reduced.

To reveal the reason for the impaired release of infectious virus from MV9G cells, we performed a comparative TEM analysis of TB40/E infected MV9G cells at 5 d p.i. HFF served as reference cell type since HCMV replication in fibroblasts has been well characterized on the ultrastructural level [37,38]. Samples were prepared by high-pressure freezing, freeze substitution, Epon embedding and ultrathin sectioning.

**Fig 5:**
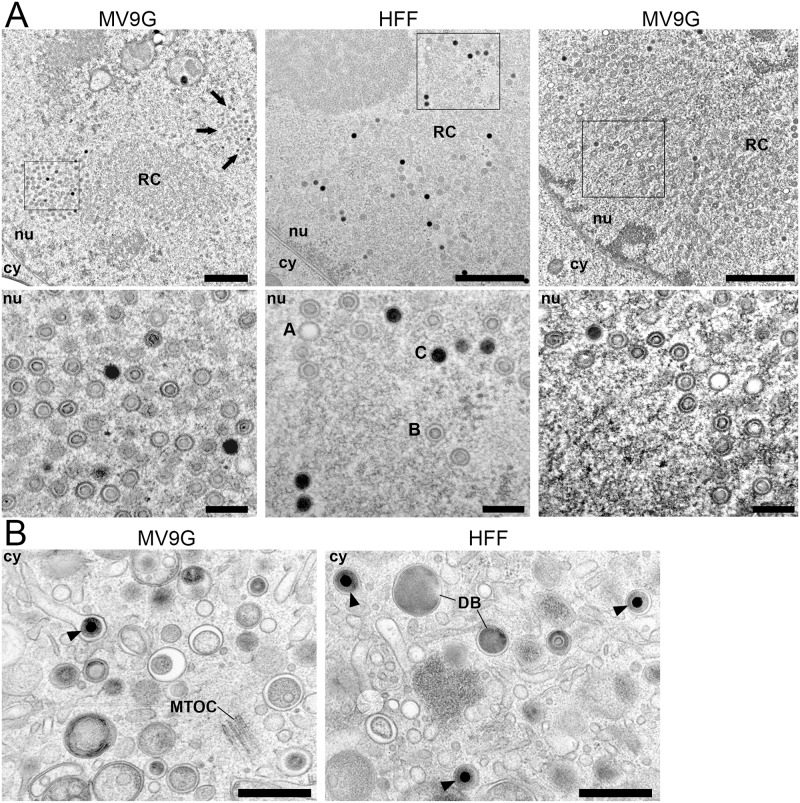
The production of infectious HCMV virions in MV9G cells is probably impaired during the nuclear stage of virion morphogenesis. (A) MV9G cells and HFF were infected with TB40/E and processed for transmission electron microscopy at 5 d p.i. Top row: The representative overviews show the distribution of nuclear capsids within the nucleoplasm of MV9G and HFF cells. Boxed areas are depicted in a higher magnification in the bottom row. Arrows in the left image point at a second patch of nuclear capsids in the MV9G cell nucleus. cy cytoplasm, nu nucleus. RC replication compartment. Scale bars: 1 μm. Bottom row: Higher magnification of the indicated areas. Three different capsid stages (A, B and C capsids) can be distinguished. Scale bars: 200 nm. (B) MV9G cells and HFF were infected with TB40/E and processed for transmission electron microscopy at 5 d p.i. The area of the cytoplasmic viral assembly complex was examined for virions (arrowheads). Representative images are shown. cy, cytoplasm; MTOC, microtubule organizing center; DB, dense bodies. Scale bars: 500 nm.

First, the nuclear stage of HCMV morphogenesis in at least 25 MV9G cells and 14 HFF was ultrastructurally visualized. The majority of MV9G nuclei exhibited a capsid distribution different from that in HFF (Fig 5A, left). The capsids in all HFF nuclei loosely accumulated in the periphery of areas with slightly higher electron density and a rougher appearance (Fig 5A, middle), most likely representing viral replication compartments (RCs), which are sites of viral DNA replication [54]. In contrast, capsids in MV9G nuclei rather accumulated in densely packed patches (Fig 5A, left). Those patches were irregularly distributed throughout the nucleus, however, most of them were found in close proximity to putative RCs. Interestingly, some of the MV9G nuclei showed a second type of capsid distribution (Fig 5A, right), which resembled that in fibroblasts (compare the two capsid distribution types to the MCP staining in Fig 4A). Independent of the type of capsid distribution, all MV9G nuclei exhibited an altered ratio of the three capsid stages (A capsid, B capsids and C capsids): Compared to fibroblast nuclei, all MV9G nuclei exhibited less DNA-containing C capsids per DNA-lacking B capsid (Fig 5A bottom). However, there was no obvious difference in the structure of capsids in MV9G and HFF nuclei. Examination of the cytoplasm of MV9G cells revealed the presence of DNA-containing C capsids in the viral assembly compartment (Fig 5B) which is the site of secondary envelopment [37,55], the final step in virion maturation. In addition, particles containing capsids of different stages as well as particles in intermediate stages of secondary envelopment [37] were detected in the cytoplasmic viral assembly compartment of infected MV9G cells as well as in HFF (data not shown). [37,55]Together, this indicates that the nuclear capsids are able to egress from the nucleus of MV9G cells and to obtain a viral envelope, which is the prerequisite for the observed release of infectivity (Fig 4C).

**Fig 6:**
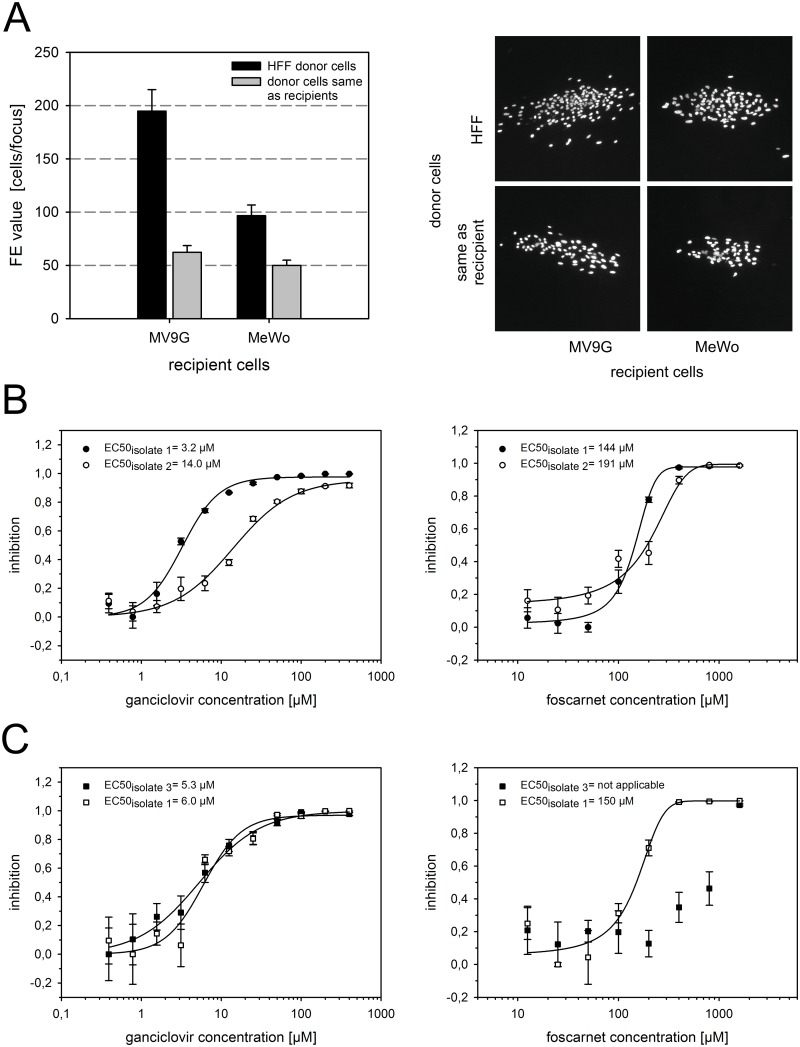
MV9G cells allow focal growth of HCMV and are suitable for drug resistance assays. (A) MV9G or MeWo cells were co-cultured with infected cells, either HFF or homotypic cells as indicated, for 7 days. Infected cells were detected by immunostaining of viral immediate early antigens and the focus expansion (FE) value (left) was determined by counting the number of cells per focus as described [42]. The graph integrates data from three independent experiments. Statistical significance was assessed by student’s t-test. Example images of immunostained foci are shown (right). (B-C) MV9G cells were incubated with HFF cultures infected with clinical HCMV isolates for two days. The cultures were exposed to serial dilutions of either GCV (left) or FOS (right) and the luciferase activity was measured. The drug resistance assays were performed in quadruplicates. A sigmoidal dose response curve was fitted onto the normalized inhibition values to determine the effective drug concentration yielding 50% inhibition (EC50). Error bars: SEM.

Since HCMV could replicate in MV9G and MeWo cells, we asked whether the virus is able to spread efficiently from cell to cell in culture. To this end, co-cultures of infected HFF (donor) and uninfected (recipient) cells (MV9G or MeWo, respectively) were prepared and the size of the foci formed after 7 days was analyzed by immunofluorescence staining of viral immediate early antigens. In both MV9G and MeWo cell cultures, discrete foci were observed (Fig 6A and 6B). The foci formed in MV9G cultures (mean focus size = 195 cells/focus ± 20 SEM) were about twofold larger than the foci in MeWo cultures (mean focus size = 97 cells/focus ± 10 SEM) (Fig 6A). With HFF as donor cells, it cannot be excluded that all infected recipient cells received their virus directly from the initially shedding HFF cell rather than via a second round of infection from a MV9G or MeWo cell, respectively. We therefore included MV9G and MeWo cells as donor cells in the experiment, so that the donor and recipient cells would be of the same cell type. Again, discrete foci were formed by both cell types (Fig 6A and 6B); yet, the focus size was significantly lower with homotypic donor cells compared to foci initiated by HFF donor cells (mean focus size in MV9G or MeWo cells 62 ± 6 or 50 ± 5 cells/focus, respectively) (Fig 6A).

A possible application of MV9G cells is the use in diagnostic assays with clinical samples where the luciferase reporter gene allows rapid and straightforward quantification. For proof-of-principle, we performed a drug resistance test in HFF in 96-well plates similar to a standard plaque reduction assay [5]. HFF infected with recent clinical isolates were cultured with increasing concentrations of either ganciclovir (GCV) or foscarnet (FOS). MV9G cells were added for two days at the end of the incubation period in order to allow infection of the reporter cells and activation of the reporter gene. For this assay, we chose isolates with a known wild-type or clinically relevant resistance-conferring genotype of the viral protein kinase UL97 and the viral DNA polymerase UL54 and that were previously characterized phenotypically by the classical plaque reduction assay. Isolate 1 does not contain any known resistance-conferring mutations in either gene, UL97 or UL54, and was therefore used as wild-type reference. Isolate 2 carries a H520Q mutation in UL97 that is described to be amongst the most frequent UL97 mutations in patients receiving GCV as initial therapy [4]. UL54 of isolate 2 appeared to be wild-type by genotype. Isolate 3 has an A809V mutation in UL54 linked to increased resistance against FOS [4] but no resistance-associated mutation in UL97. According to the genotypic data and our previous analysis of these isolates with the classical plaque reduction assay (data not shown), isolate 2 is expected to be resistant to GCV and sensitive to FOS while isolate 3 should be resistant to FOS and sensitive to GCV. In the modified drug resistance test with MV9G cells, all three isolates were able to induce luciferase activity in MV9G cells (Fig 6B and 6C) showing that, in principle, MV9Gs are susceptible and the reporter gene is responsive to clinical isolates of HCMV. According to the expectations, FOS was similarly effective against isolates 1 and 2 with an EC50 of 144 μM or 191 μM, respectively, and isolate 2 was >4.3 fold less sensitive to GCV than isolate 1 (Fig 6B). GCV inhibited isolate 1 and 3 to a similar extent (EC50 of 6 μM versus 5.3 μM, respectively) (Fig 6C). Isolate 3 behaved resistant to FOS in the luciferase assay with an estimated EC50 of >500 μM compared to an EC50 of isolate 1 of 150 μM (Fig 6C). The exact EC50 of FOS could not be determined for isolate 3 because fitting of a sigmoidal dose response curve to the data failed. Together, these results demonstrate that MV9G cells are applicable to clinical HCMV isolates and can be useful in drug resistance tests.

**Fig 7:**
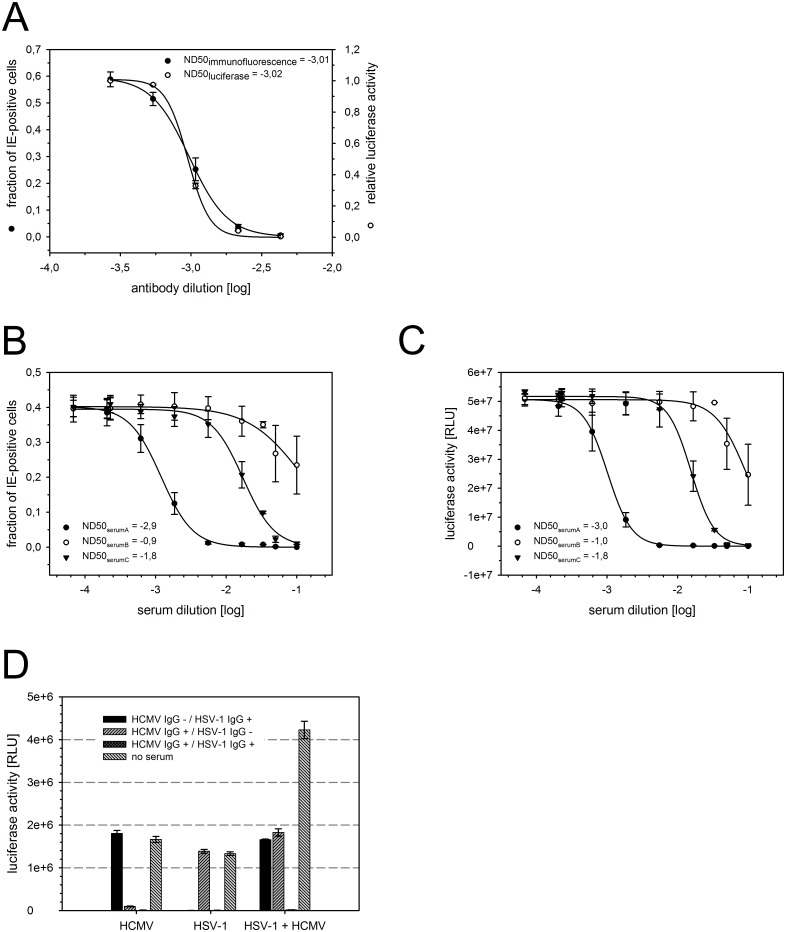
The luciferase reporter can be applied to determine the neutralization capacity of HCMV antibodies and sera as well as to comparative analysis of HSV-1 and HCMV. (A-C) HCMV TB40/E was incubated with serial dilutions of glycoprotein B (gB) antibody (A) or human sera (B-C) prior to infection. Infection efficiency was determined by immunostaining of viral immediate early antigens at 1 d p.i. and luciferase activity was measured in cell lysates harvested at 2 d p.i. (A) The infection efficiency and luciferase signal obtained without antibody was 71% or 5.3e+7 RLU, respectively. (B-C) Serum A, filled circle; serum B, open circle; serum C, filled triangle. The mean values of at least three independent experiments are shown. ND50: 50% neutralization dose. (D) HSV-1 or HCMV or a mixture of both were preincubated with three human sera of known serostatus before infection of MV9Gs. Luciferase activity was measured in cell lysates at 1 d p.i. Error bars: SEM.

As described above, we found that the activation of the reporter gene in MV9G cells requires *de novo* expression of the viral immediate early transactivators and that the mere presence of virion components is not sufficient. Therefore, another putative application of this reporter cell line is the evaluation of the neutralization capacity of HCMV antibodies or patient-derived sera. First, we tested whether inhibition of HCMV entry by an antibody against the viral fusogen glycoprotein B (gB) can accurately be displayed by the reporter. HCMV TB40/E was incubated with serial dilutions of gB antibody for 1 h. The mixture was added to MV9G cells for 2 h to allow infection. Parallel cultures were prepared (i) for analysis of infection efficiency by immunostaining of viral immediate early antigens at 1 d p.i. and (ii) for measurement of luciferase activity in cell lysates at 2 d p.i. Increasing amounts of gB antibody led to reduced infection and the reporter gene activity accurately followed the infection efficiency (Fig 7A). Similar 50% neutralization doses were obtained with both methods (ND50_immunofluorescence_ = -3.01; ND50_luciferase_ = -3.02).

The same experimental setup was subsequently applied to test three patient-derived sera with known high (IgG index >10, arbitrarily defined), medium (IgG index ≥1.1) or low (IgG index <0.9) antibody concentration (CMV-IgG-ELISA PKS, Medac, Germany) (IgG index_serum A_ = 15.7; IgG index_serum B_ = 0.18; IgG index_serum C_ = 3.73). Again, the infection efficiency expressed as the fraction of viral immediate early antigen-expressing cells (Fig 7B) was accurately displayed by the luciferase activity in replicate cultures (Fig 7C). The serum dilution [log] leading to 50% neutralization (ND50) was almost identical in both sets of data (ND50 of serum A: -2.9 and -3.0; serum B: -0.9 and -1.0; serum C: -1.8 and -1.8) (Fig 7B and 7C). Together, the MV9G reporter cell line is a suitable assay system for determining the neutralization capacity of HCMV antibodies and sera.

In order to test whether different herpesviruses can be comparatively analyzed side-by-side in MV9Gs, HSV-1 and HCMV as well as a mixture of both viruses were preincubated with three human sera differing in their serostatus. MV9G cells were infected with the serum/virus-solution and luciferase activity was analyzed. In absence of human serum, both viruses induced luciferase activity individually as well as when combined in the same culture (Fig 7D). In cultures where HSV-1 and HCMV were simultaneously present, the luciferase activity was approximately doubled which is in agreement with the twofold increase in the total amount of virus. Both viruses were efficiently neutralized by the dual seropositive and by the respective single seropositive human serum as indicated by a failure of these combinations to induce luciferase activity. These results prove that herpesviruses of different herpesvirinae subfamilies can be analyzed in MV9G cells not only in a single comparative experiment but even simultaneously in the same culture wells.

## Discussion

### Susceptibility and permissiveness to HCMV

The aim of this work was to test and possibly adopt a cell-based reporter system in a physiologically relevant human cell type for the quantification of HCMV infection. To this end, the heterologous reporter cell line MV9G was evaluated since it is based on an epithelial cell type with a high probability of being susceptible to HCMV infection. Moreover, the reporter gene had been shown previously to be responsive not only to VZV but also to HSV-1 [22,25]. Indeed, we found that MV9G cells as well as their parental cell line MeWo are both susceptible to HCMV infection and, in contrast to most permanent cell lines tested so far [24,50–53], support viral replication and spread. Replication of viral DNA and viral antigen expression appeared to occur at similar velocity and levels as in prototypic fibroblasts or endothelial cells. Albeit lower than in HFF or HEC-LTT cells, the titers of released infectivity that are reached are probably sufficient for most applications which qualifies MV9G and MeWo cells as a comprehensive cell culture system for HCMV.

The reduced release of infectious progeny virus into the supernatant and diminished viral spread in the cell culture is probably due to an impaired DNA encapsidation step and/or to a different structure of nuclear replication sites. It will be interesting to analyze the nuclear stage of replication in MV9G or MeWo cells in greater detail as this might help to better understand the mechanism of viral replication and the contribution of cellular factors.

### Responsiveness of the heterologous reporter gene to HCMV

The luciferase reporter gene was shown before to respond to expression of the VZV major transactivator IE62 in absence of infection [22]. Similarly, the activation of the reporter gene during HCMV infection requires successful viral entry including immediate early antigen expression. This is supported by several findings: Firstly, poorly epitheliotropic viruses (Towne, TB40/F) activate the reporter only mildly and the level of reporter activity corresponds to the fraction of IE-positive cells rather than to the amount of virions in the inoculum. Secondly, neutralizing antibodies/sera inhibit reporter gene expression in a dose-dependent manner. Thirdly, reporter gene expression in TB40/E-infected cultures but not in HSV-1-infected cultures is prevented if the cells are loaded with IE siRNA prior to infection. Fourthly, transient expression of HCMV IE antigens is sufficient to induce luciferase activity. Together, these data indicate that the reporter gene is selectively activated in HCMV infected cells in a dose-dependent manner.

Our finding that the reporter gene in MV9G cells is responsive to HCMV infection is not trivial because of two aspects. First, there is to our knowledge no orthologue of VZV IE62 known in beta- or gammaherpesviruses while HSV-1 –that is a close relative of VZV and that we and others found to activate the reporter gene [22,25]—encodes a functional orthologue of VZV IE62 termed ICP4 [48,56–58]. Thus, it was unobvious that the reporter gene responds to infection with herpesviruses from other subfamilies. Second, our data stands in contrast to the observation by Wang and colleagues who mentioned that they did not detect luciferase activity upon HCMV infection [22]. Details of their experiment are unfortunately not given but the authors mention that HCMV strain Towne was used. The strain Towne is only poorly epitheliotropic and infects MV9G cells at much lower efficiency than fibroblasts (relative tropism 1.1%) which is a likely explanation for the observed difference. In our hands, the luciferase activity in MV9G cells infected with Towne or with another poorly epitheliotropic strain, TB40/F, were detectable at levels comparable to the fully epitheliotropic strain TB40/E if corrected for the infection efficiency. Therefore, MV9G cells might be useful even to study poorly epitheliotropic virus strains if just larger amounts of virus are added compared to highly epitheliotropic strains.

### Applications and benefit of the MV9G reporter cell line

Our findings not only indicate that MV9G cells are applicable to study different aspects of HCMV biology but they also open a range of potential diagnostic applications. Diagnostic approaches for phenotypic analysis of clinical samples such as detection of infectivity, drug resistance testing or analysis of neutralization of clinical isolates by antibodies/sera require a test system that can sense and quantify unlabeled virus. Phenotypic assays such as the classical plaque reduction assay and quantification methods such as immunoblotting are often laborious and the validity of their results depends strongly on the skills of experienced personnel. A reporter cell line that allows objective, robust and straightforward assays is therefore desirable and can help to reduce inconveniences of the existing test systems and to increase throughput.

As a proof-of-principle, we have shown that MV9G cells can be used to test the sensitivity or resistance of clinical isolates against two drugs, GCV and FOS. The heterologous luciferase reporter did not only respond to HCMV but accurately displayed the known resistance phenotype of previously characterized clinical isolates. As a second application, entry inhibition by gB antibody and neutralizing sera was evaluated using MV9G cells and the luciferase activity accurately mirrored the infection efficiency. Together, these data demonstrate that MV9G cells are suitable to study the effects of antiviral agents acting in different phases of the HCMV replication cycle. Furthermore, MV9G cells have been shown to be suitable for high-throughput screening of potential antiviral compounds against VZV [59] which might be useful to identify novel HCMV inhibitors in the future.

As mentioned above, immortalized cell lines often allow expression of immediate early antigens of HCMV but viral replication is normally blocked at a later step of the replicative cycle [24,50–53]. In line with this, Fukui et al. mention that their reporter cell line established in the background of a human glioma cell line is not suitable to analyze inhibition of any process after transactivation of the reporter’s TLR4 early promoter [16]. This limitation also applies to the other existing reporter cell lines including those generated in the background of mink lung cells or CHO cells [17–20] and previous attempts to generate a reporter cell line with full permissiveness to HCMV failed [20]. Therefore, a major advantage of the MV9G cells over other HCMV reporter cell lines is that they are productively infected at least to some extent.

The permissiveness of the MV9G cells to HCMV could be particularly useful with regard to clinical isolates of HCMV. HCMV isolates tend to lose their broad cell tropism quickly when they are passaged in fibroblasts which is mainly due to mutations in genes of the UL128 locus [43,46,47,60]. However, they would be expected to remain genetically rather stable in MV9G/MeWo cells because of the epithelial origin of the cells. Although this remains to be tested, it might in the future open the possibility to stably maintain HCMV from clinical samples and rapidly examine the virus in the same cell type without a detour via fibroblasts.

Where tested, the previously used HCMV reporter cell lines did not respond to infection by other human herpesviruses such as HSV-1 and HSV-2 [17] or VZV and human herpesvirus type 6 [16]. In contrast, MV9G cells have been used before to quantitate infection with two alphaherpesviruses (VZV and HSV-1) [22,25,59]. As demonstrated by these studies and the data presented here, MV9G cells enable comparative analyses of human herpesviruses of different herpesvirinae subfamilies in the same assay system and even as a virus mixture in the same culture which is another unique advantage over other available HCMV reporter cell lines.
